# The land crab *Johngarthia planata* (Stimpson, 1860) (Crustacea, Brachyura, Gecarcinidae) colonizes human-dominated ecosystems in the continental mainland coast of Mexico

**DOI:** 10.3897/BDJ.2.e1161

**Published:** 2014-07-08

**Authors:** Robert Perger

**Affiliations:** † Coleccion Boliviana de Fauna, La Paz, Bolivia

**Keywords:** Brachyura, dispersal, distribution, East Pacific, Mexico, new record

## Abstract

The land crab *Johngarthia planata* (Stimpson, 1860) has been reported from the Baja California Peninsula and several oceanic islands in the Eastern Pacific as well as inshore islands of the Mexican, Costa Rican and Colombian coast. However, the species has not been observed on the continental mainland, as it is likely that the high diversity of terrestrial predators/competitors make the establishment of mainland populations nearly impossible. In this contribution, several new records of this species that have been observed in urban areas along the continental Pacific coast of Mexico are reported. These records demonstrate that the presence of humans does not necessarily have a negative impact on land crab species. Indeed, the presence of humans may actually discourage the presence of native crab predators/competitors and hence increase the likelihood of a successful mainland settlement of land crab species that are otherwise island and peninsula restricted. The presence of *Johngarthia planata* is ecologically relevant for coastal forests because gecarcinid crabs significantly influence plant recruitment and *Johngarthia planata* is considerably larger than the mainland species *Gecarcinus quadratus*.

## Introduction

Macroevolutionary transitions between marine and non-marine habitats are uncommon and rarely lead to long-term success in the invaded habitat (Vermeij and Dudley 2000). This is largely due to terrestrial niches being occupied by groups with a longer evolutionary history in these habitats as well as the marine invaders' lack of traits withwhich to successfully confront the differences in terrestrial selection regimes (Vermeij and Dudley 2000).

However, while their larvae still develop in marine habitats, adults of the crab family Gecarcinidae have successfully conquered island habitats, where they play an important ecological role (Lindquist et al. (2009) for a review). Species of the *Gecarcinus*-group (including *Johngarthia*, *Gecarcinus* and *Gecarcoidea* (Türkay 1987) are dominant through sheer numbers, on some islands their mass exceeds the total mass of animals reported in tropical rain forests in Costa Rica and the central Amazon (Lindquist et al. 2009) and they may occupy the top of the energy pyramid (Burggren and McMahon 1988).

Nevertheless, despite their success on islands and possible dispersal in their larval stage via sea currents, most species of the *Gecarcinus*-group are absent from continental mainland habitats (Türkay 1987; see Paulay and Starmer 2011). One reason for this may be the predictable mass migrations to the coast for larval release (e.g. Linnaeus 1758, Hicks 1985, Hartnoll 2011), which may render species of this group especially vulnerable to terrestrial predators, therefore limiting dispersion and the chances of success on the continental mainland. The negative impact on populations exerted by e.g. terrestrial vertebrates is supported by observed population declines or extinctions after island settlements by humans, which often actively hunt land crabs, or after the anthropogenic introduction of crab predators/competitors such as rats, feral cats and pigs (Paulay and Starmer 2011).

*Johngarthia planata* (Stimpson, 1860) appears to be a textbook example for the group's failure to establish itself on the continental mainland despite the advantages of ecological flexibility and favorable larval dispersion patterns (reviewed by Perger et al. 2013). *Johngarthia planata* occurs on the Baja California Peninsula and several oceanic islands in the Eastern Pacific as well as on inshore islands of the Mexican, Costa Rican and Colombian coasts. This wide presence is likely the result of amplified dispersal via coastal currents (Perger et al. 2013). However, likely due to the large diversity of terrestrial predators/competitors, *Johngarthia planata* has not been observed on continental mainland locations until now (Perger et al. 2013).

In this study I report the first continental mainland records for *Johngarthia planata* and discuss the possibility that human presence might actually facilitate the mainland settlement of such a species.

## Materials and methods

The present study encompasses the Pacific coast from Mexico to Colombia. Following Grismer (2000) and Riddle et al. (2000) the Baja California peninsula is treated separately to the rest of Mexico, which is referred to throughout the text as “Mexican mainland” or “continental mainland” respectively. To investigate the mainland presence/absence patterns of *Johngarthia planata* I performed an Internet search via Google.com (search function “images”) and flickr.com. Flickr is an image hosting web site that is reported to host more than 6 billion photographs. Users uploading their photos to Flickr are able to add key words (“tags”) that facilitate a search on Flickr and also in web search services such as Google and Yahoo.  I searched with various combinations of the key words “crab” or “cangrejo” (Spanish for crab) and “Pacific, Mexico, Guatemala, El Salvador, Nicaragua, Costa Rica, Panama, Colombia”; and all regions of the respective countries with access to the Pacific coast. I then considered for evaluation only individuals on photographs from locations that were confirmed by the photographers and demonstrated clearly visible characteristics of *Johngarthia planata* (see Perger et al. 2013).

The following abbreviations are used in the text: LACM – Natural History Museum of Los Angeles County, Los Angeles, California; MNHN – Muséum National d’Histoire Naturelle, Paris, France; MZ-UCR – Museo de Zoología, Universidad de Costa Rica, San José, Costa Rica; USNM – National Museum of Natural History, Smithsonian Institution, Washington, D.C.

## Taxon treatments

### Johngarthia planata

(Stimpson, 1860)

#### Materials

- **Type status:** Other material. **Occurrence:** recordedBy: Robert Perger; individualCount: 2; sex: 1 male, 1 female; reproductiveCondition: egg-bearing female; behavior: running, agonistic display, pinching, producing bubbles in mouth area; **Taxon:** scientificName: *Johngarthia planata* Stimpson, 1860; namePublishedIn: Stimpson W (1860) Notes on North American Crustacea, in the museum of the Smithsonian Institution, No II. Annals of the Lyceum of Natural History of New York 7: 176–246.; order: Decapoda; family: Gecarcinidae; genus: Johngarthia; specificEpithet: planata; scientificNameAuthorship: W. Stimpson 1860; **Location:** waterBody: East Pacific; island: Caño Island; country: Costa Rica; stateProvince: Puntarenas; verbatimLocality: Caño Island; verbatimElevation: 39 m; decimalLatitude: 8.70861111111111; decimalLongitude: -83.88916666666667; **Identification:** identificationID: Robert Perger; **Event:** samplingProtocol: manual search; samplingEffort: 2 hours; eventDate: 2011-02-25
- **Type status:** Other material. **Occurrence:** recordedBy: Robert Perger; individualCount: 14; sex: juveniles; **Taxon:** scientificName: *Johngarthia planata* Stimpson, 1860; namePublishedIn: Stimpson W (1860) Notes on North American Crustacea, in the museum of the Smithsonian Institution, No II. Annals of the Lyceum of Natural History of New York 7: 176–246.; order: Decapoda; family: Gecarcinidae; genus: Johngarthia; specificEpithet: planata; scientificNameAuthorship: W. Stimpson 1860; **Location:** waterBody: East Pacific; island: Nairita Island; country: Costa Rica; stateProvince: Puntarenas; verbatimLocality: Nairita Island; verbatimElevation: 1 m; decimalLatitude: 8.67; decimalLongitude: -83.71777777777778; **Identification:** identificationID: Robert Perger; **Event:** samplingProtocol: manual search; samplingEffort: 4 hours; eventDate: 2011-03-17; **Record Level:** institutionCode: MZ-UCR
- **Type status:** Other material. **Occurrence:** recordedBy: F. Joyce; individualCount: 2; sex: males; **Taxon:** scientificName: *Johngarthia planata* Stimpson, 1860; namePublishedIn: Stimpson W (1860) Notes on North American Crustacea, in the museum of the Smithsonian Institution, No II. Annals of the Lyceum of Natural History of New York 7: 176–246.; order: Decapoda; family: Gecarcinidae; genus: Johngarthia; specificEpithet: planata; scientificNameAuthorship: W. Stimpson 1860; **Location:** waterBody: East Pacific; island: Colorada Island; country: Costa Rica; stateProvince: Guanacaste; verbatimLocality: Colorada Island; verbatimElevation: 13 m; decimalLatitude: 10.846111111111112; decimalLongitude: -85.8611111111111; **Identification:** identificationID: Robert Perger; **Event:** eventDate: 2000-10-20; **Record Level:** institutionCode: MZ-UCR
- **Type status:** Other material. **Occurrence:** recordedBy: A. Anthony; individualCount: 1; sex: male; **Taxon:** scientificName: *Johngarthia planata* Stimpson, 1860; namePublishedIn: Stimpson W (1860) Notes on North American Crustacea, in the museum of the Smithsonian Institution, No II. Annals of the Lyceum of Natural History of New York 7: 176–246.; order: Decapoda; family: Gecarcinidae; genus: Johngarthia; specificEpithet: planata; scientificNameAuthorship: W. Stimpson 1860; **Location:** waterBody: East Pacific; island: Socorro Island; country: Mexico; verbatimLocality: Socorro Island; **Record Level:** institutionCode: MNHN
- **Type status:** Other material. **Occurrence:** individualCount: 2; sex: males; **Taxon:** scientificName: *Johngarthia planata* Stimpson, 1860; namePublishedIn: Stimpson W (1860) Notes on North American Crustacea, in the museum of the Smithsonian Institution, No II. Annals of the Lyceum of Natural History of New York 7: 176–246.; order: Decapoda; family: Gecarcinidae; genus: Johngarthia; specificEpithet: planata; scientificNameAuthorship: W. Stimpson 1860; **Location:** waterBody: East Pacific; island: Socorro Island; country: Mexico; verbatimLocality: Socorro Island; **Identification:** identificationID: John Garth; **Event:** eventDate: 1971-02-16; **Record Level:** institutionCode: LACM
- **Type status:** Other material. **Occurrence:** recordedBy: A. Anthony; individualCount: 1; sex: male; **Taxon:** scientificName: *Johngarthia planata* Stimpson, 1860; namePublishedIn: Stimpson W (1860) Notes on North American Crustacea, in the museum of the Smithsonian Institution, No II. Annals of the Lyceum of Natural History of New York 7: 176–246.; order: Decapoda; family: Gecarcinidae; genus: Johngarthia; specificEpithet: planata; scientificNameAuthorship: W. Stimpson 1860; **Location:** waterBody: East Pacific; island: Socorro Island; country: Mexico; verbatimLocality: Socorro Island; **Record Level:** institutionCode: USNM
- **Type status:** Other material. **Occurrence:** individualCount: 2; sex: males; **Taxon:** scientificName: *Johngarthia planata* Stimpson, 1860; namePublishedIn: Stimpson W (1860) Notes on North American Crustacea, in the museum of the Smithsonian Institution, No II. Annals of the Lyceum of Natural History of New York 7: 176–246.; order: Decapoda; family: Gecarcinidae; genus: Johngarthia; specificEpithet: planata; scientificNameAuthorship: W. Stimpson 1860; **Location:** waterBody: East Pacific; island: San Benedicto Island; country: Mexico; verbatimLocality: San Benedicto Island; **Identification:** identificationID: John Garth; **Record Level:** institutionCode: LACM
- **Type status:** Other material. **Occurrence:** recordedBy: A. Anthony; individualCount: 1; sex: male; **Taxon:** scientificName: *Johngarthia planata* Stimpson, 1860; namePublishedIn: Stimpson W (1860) Notes on North American Crustacea, in the museum of the Smithsonian Institution, No II. Annals of the Lyceum of Natural History of New York 7: 176–246.; order: Decapoda; family: Gecarcinidae; genus: Johngarthia; specificEpithet: planata; scientificNameAuthorship: W. Stimpson 1860; **Location:** waterBody: East Pacific; island: San Benedicto Island; country: Mexico; verbatimLocality: San Benedicto Island; **Record Level:** institutionCode: USNM
- **Type status:** Other material. **Occurrence:** recordedBy: Léon Diguet; individualCount: 1; sex: male; **Taxon:** scientificName: *Johngarthia planata* Stimpson, 1860; namePublishedIn: Stimpson W (1860) Notes on North American Crustacea, in the museum of the Smithsonian Institution, No II. Annals of the Lyceum of Natural History of New York 7: 176–246.; order: Decapoda; family: Gecarcinidae; genus: Johngarthia; specificEpithet: planata; scientificNameAuthorship: W. Stimpson 1860; **Location:** waterBody: East Pacific; country: Mexico; verbatimLocality: Baja California; **Record Level:** institutionCode: MNHN
- **Type status:** Other material. **Occurrence:** recordedBy: Edward William Nelson; Edward Alphonso Goldman; individualCount: 1; sex: male; **Taxon:** scientificName: *Johngarthia planata* Stimpson, 1860; namePublishedIn: Stimpson W (1860) Notes on North American Crustacea, in the museum of the Smithsonian Institution, No II. Annals of the Lyceum of Natural History of New York 7: 176–246.; order: Decapoda; family: Gecarcinidae; genus: Johngarthia; specificEpithet: planata; scientificNameAuthorship: W. Stimpson 1860; **Location:** waterBody: East Pacific; country: Mexico; verbatimLocality: María Cleofas Island; **Event:** eventDate: 1897-05-30; **Record Level:** institutionCode: USNM
- **Type status:** Other material. **Occurrence:** recordedBy: Conrad Limbaugh; individualCount: 4; sex: males; **Taxon:** scientificName: *Johngarthia planata* Stimpson, 1860; namePublishedIn: Stimpson W (1860) Notes on North American Crustacea, in the museum of the Smithsonian Institution, No II. Annals of the Lyceum of Natural History of New York 7: 176–246.; order: Decapoda; family: Gecarcinidae; genus: Johngarthia; specificEpithet: planata; scientificNameAuthorship: W. Stimpson 1860; **Location:** waterBody: East Pacific; country: France; verbatimLocality: Clipperton Island; **Identification:** identificationID: John Garth; **Event:** eventDate: 1958-09-12; **Record Level:** institutionCode: LACM
- **Type status:** Other material. **Occurrence:** individualCount: 1; sex: male; **Taxon:** scientificName: *Johngarthia planata* Stimpson, 1860; namePublishedIn: Stimpson W (1860) Notes on North American Crustacea, in the museum of the Smithsonian Institution, No II. Annals of the Lyceum of Natural History of New York 7: 176–246.; order: Decapoda; family: Gecarcinidae; genus: Johngarthia; specificEpithet: planata; scientificNameAuthorship: W. Stimpson 1860; **Location:** waterBody: East Pacific; country: France; verbatimLocality: Clipperton Island; **Identification:** identificationID: Danièle Guinot; **Record Level:** institutionCode: MNHN
- **Type status:** Other material. **Occurrence:** recordedBy: J. Arnheim; individualCount: 2; sex: 1 male, 1 female; **Taxon:** scientificName: *Johngarthia planata* Stimpson, 1860; namePublishedIn: Stimpson W (1860) Notes on North American Crustacea, in the museum of the Smithsonian Institution, No II. Annals of the Lyceum of Natural History of New York 7: 176–246.; order: Decapoda; family: Gecarcinidae; genus: Johngarthia; specificEpithet: planata; scientificNameAuthorship: W. Stimpson 1860; **Location:** waterBody: East Pacific; country: France; verbatimLocality: Clipperton Island; **Record Level:** institutionCode: USNM
- **Type status:** Other material. **Occurrence:** recordedBy: María Elena Valencia; individualCount: 1; sex: female; **Taxon:** scientificName: *Johngarthia planata* Stimpson, 1860; namePublishedIn: Stimpson W (1860) Notes on North American Crustacea, in the museum of the Smithsonian Institution, No II. Annals of the Lyceum of Natural History of New York 7: 176–246.; order: Decapoda; family: Gecarcinidae; genus: Johngarthia; specificEpithet: planata; scientificNameAuthorship: W. Stimpson 1860; **Location:** waterBody: East Pacific; country: Panama; verbatimLocality: Iguana Island; **Identification:** identificationID: Robert Perger; **Event:** year: 2006; habitat: beach; **Record Level:** type: Photograph; rightsHolder: María Elena Valencia
- **Type status:** Other material. **Occurrence:** recordedBy: Julio Larish; individualCount: 1; sex: juvenile; **Taxon:** scientificName: *Johngarthia planata* Stimpson, 1860; namePublishedIn: Stimpson W (1860) Notes on North American Crustacea, in the museum of the Smithsonian Institution, No II. Annals of the Lyceum of Natural History of New York 7: 176–246.; order: Decapoda; family: Gecarcinidae; genus: Johngarthia; specificEpithet: planata; scientificNameAuthorship: W. Stimpson 1860; **Location:** waterBody: East Pacific; country: Panama; verbatimLocality: Iguana Island; **Identification:** identificationID: Robert Perger; **Event:** year: 2013; habitat: beach; **Record Level:** type: Photograph; rightsHolder: Julio Larish
- **Type status:** Other material. **Occurrence:** recordedBy: Lisa Brettschneider; individualCount: 1; **Taxon:** scientificName: *Johngarthia planata* Stimpson, 1860; namePublishedIn: Stimpson W (1860) Notes on North American Crustacea, in the museum of the Smithsonian Institution, No II. Annals of the Lyceum of Natural History of New York 7: 176–246.; order: Decapoda; family: Gecarcinidae; genus: Johngarthia; specificEpithet: planata; scientificNameAuthorship: W. Stimpson 1860; **Location:** waterBody: East Pacific; country: Mexico; stateProvince: Sinaloa; verbatimLocality: Mazatlán; **Identification:** identificationID: Robert Perger; **Event:** year: 2008; habitat: beach; **Record Level:** type: Photograph; rightsHolder: Lisa Brettschneider
- **Type status:** Other material. **Occurrence:** recordedBy: Lisa Johnston; individualCount: 1; **Taxon:** scientificName: *Johngarthia planata* Stimpson, 1860; namePublishedIn: Stimpson W (1860) Notes on North American Crustacea, in the museum of the Smithsonian Institution, No II. Annals of the Lyceum of Natural History of New York 7: 176–246.; order: Decapoda; family: Gecarcinidae; genus: Johngarthia; specificEpithet: planata; scientificNameAuthorship: W. Stimpson 1860; **Location:** waterBody: East Pacific; country: Mexico; stateProvince: Nayarit; verbatimLocality: Sayulita; **Identification:** identificationID: Robert Perger; **Event:** year: 2005; habitat: beach; **Record Level:** type: Photograph; rightsHolder: Lisa Johnston
- **Type status:** Other material. **Occurrence:** recordedBy: Tania Beagley-Brown; individualCount: 1; **Taxon:** scientificName: *Johngarthia planata* Stimpson, 1860; namePublishedIn: Stimpson W (1860) Notes on North American Crustacea, in the museum of the Smithsonian Institution, No II. Annals of the Lyceum of Natural History of New York 7: 176–246.; order: Decapoda; family: Gecarcinidae; genus: Johngarthia; specificEpithet: planata; scientificNameAuthorship: W. Stimpson 1860; **Location:** waterBody: East Pacific; country: Mexico; stateProvince: Nayarit; verbatimLocality: Sayulita; **Identification:** identificationID: Robert Perger; **Event:** year: 2007; habitat: on roof of house; **Record Level:** type: Photograph; rightsHolder: Tania Beagley-Brown
- **Type status:** Other material. **Occurrence:** recordedBy: Daniel Brewer; individualCount: 2; sex: 1 male, 1 female; **Taxon:** scientificName: *Johngarthia planata* Stimpson, 1860; namePublishedIn: Stimpson W (1860) Notes on North American Crustacea, in the museum of the Smithsonian Institution, No II. Annals of the Lyceum of Natural History of New York 7: 176–246.; order: Decapoda; family: Gecarcinidae; genus: Johngarthia; specificEpithet: planata; scientificNameAuthorship: W. Stimpson 1860; **Location:** waterBody: East Pacific; country: Mexico; stateProvince: Nayarit; verbatimLocality: Sayulita; **Identification:** identificationID: Robert Perger; **Event:** year: 2010; habitat: beach; **Record Level:** type: Photograph; rightsHolder: Daniel Brewer
- **Type status:** Other material. **Occurrence:** recordedBy: Daniel Brewer; individualCount: 2; sex: 1 male, 1 female; **Taxon:** scientificName: *Johngarthia planata* Stimpson, 1860; namePublishedIn: Stimpson W (1860) Notes on North American Crustacea, in the museum of the Smithsonian Institution, No II. Annals of the Lyceum of Natural History of New York 7: 176–246.; order: Decapoda; family: Gecarcinidae; genus: Johngarthia; specificEpithet: planata; scientificNameAuthorship: W. Stimpson 1860; **Location:** waterBody: East Pacific; country: Mexico; stateProvince: Nayarit; verbatimLocality: Sayulita; **Identification:** identificationID: Robert Perger; **Event:** year: 2010; habitat: beach; **Record Level:** type: Photograph; rightsHolder: Daniel Brewer
- **Type status:** Other material. **Occurrence:** recordedBy: Madeline Milne; individualCount: 1; sex: male; **Taxon:** scientificName: *Johngarthia planata* Stimpson, 1860; namePublishedIn: Stimpson W (1860) Notes on North American Crustacea, in the museum of the Smithsonian Institution, No II. Annals of the Lyceum of Natural History of New York 7: 176–246.; order: Decapoda; family: Gecarcinidae; genus: Johngarthia; specificEpithet: planata; scientificNameAuthorship: W. Stimpson 1860; **Location:** waterBody: East Pacific; country: Mexico; stateProvince: Nayarit; verbatimLocality: Sayulita; **Identification:** identificationID: Robert Perger; **Event:** year: 2013; habitat: beach; **Record Level:** type: Photograph; rightsHolder: Madeline Milne
- **Type status:** Other material. **Occurrence:** recordedBy: Darin Williams; individualCount: 1; **Taxon:** scientificName: *Johngarthia planata* Stimpson, 1860; namePublishedIn: Stimpson W (1860) Notes on North American Crustacea, in the museum of the Smithsonian Institution, No II. Annals of the Lyceum of Natural History of New York 7: 176–246.; order: Decapoda; family: Gecarcinidae; genus: Johngarthia; specificEpithet: planata; scientificNameAuthorship: W. Stimpson 1860; **Location:** waterBody: East Pacific; country: Mexico; stateProvince: Jalisco; verbatimLocality: Mismaloya; **Identification:** identificationID: Robert Perger; **Event:** year: 2007; habitat: beach; **Record Level:** type: Photograph; rightsHolder: Darin Williams
- **Type status:** Other material. **Occurrence:** recordedBy: Derek Zoebelein; individualCount: 1; **Taxon:** scientificName: *Johngarthia planata* Stimpson, 1860; namePublishedIn: Stimpson W (1860) Notes on North American Crustacea, in the museum of the Smithsonian Institution, No II. Annals of the Lyceum of Natural History of New York 7: 176–246.; order: Decapoda; family: Gecarcinidae; genus: Johngarthia; specificEpithet: planata; scientificNameAuthorship: W. Stimpson 1860; **Location:** waterBody: East Pacific; country: Mexico; stateProvince: Colima; verbatimLocality: Manzanillo; **Identification:** identificationID: Robert Perger; **Event:** year: 2004; habitat: beach; **Record Level:** type: Photograph; rightsHolder: Derek Zoebelein
- **Type status:** Other material. **Occurrence:** recordedBy: Miguel Angel Morales; individualCount: 1; sex: male; **Taxon:** scientificName: *Johngarthia planata* Stimpson, 1860; namePublishedIn: Stimpson W (1860) Notes on North American Crustacea, in the museum of the Smithsonian Institution, No II. Annals of the Lyceum of Natural History of New York 7: 176–246.; order: Decapoda; family: Gecarcinidae; genus: Johngarthia; specificEpithet: planata; scientificNameAuthorship: W. Stimpson 1860; **Location:** waterBody: East Pacific; country: Mexico; stateProvince: Guerrero; verbatimLocality: Ixtapa; **Identification:** identificationID: Robert Perger; **Event:** year: 2012; habitat: beach; **Record Level:** type: Photograph; rightsHolder: Miguel Angel Morales
- **Type status:** Other material. **Occurrence:** recordedBy: Gustavo A. Zambrano Cabrera; individualCount: 1; sex: male; **Taxon:** scientificName: *Johngarthia planata* Stimpson, 1860; namePublishedIn: Stimpson W (1860) Notes on North American Crustacea, in the museum of the Smithsonian Institution, No II. Annals of the Lyceum of Natural History of New York 7: 176–246.; order: Decapoda; family: Gecarcinidae; genus: Johngarthia; specificEpithet: planata; scientificNameAuthorship: W. Stimpson 1860; **Location:** waterBody: East Pacific; country: Mexico; stateProvince: Oaxaca; verbatimLocality: Mazunte; **Identification:** identificationID: Robert Perger; **Event:** year: 2008; habitat: beach; **Record Level:** type: Photograph; rightsHolder: Gustavo A. Zambrano Cabrera
- **Type status:** Other material. **Occurrence:** recordedBy: Mike Gardiner; individualCount: 1; sex: male; **Taxon:** scientificName: *Johngarthia planata* Stimpson, 1860; namePublishedIn: Stimpson W (1860) Notes on North American Crustacea, in the museum of the Smithsonian Institution, No II. Annals of the Lyceum of Natural History of New York 7: 176–246.; order: Decapoda; family: Gecarcinidae; genus: Johngarthia; specificEpithet: planata; scientificNameAuthorship: W. Stimpson 1860; **Location:** waterBody: East Pacific; country: Mexico; stateProvince: Oaxaca; verbatimLocality: Escondido Beach; **Identification:** identificationID: Robert Perger; **Event:** year: 2010; habitat: beach; **Record Level:** type: Photograph; rightsHolder: Mike Gardiner
- **Type status:** Other material. **Occurrence:** recordedBy: Claudia Glechner; individualCount: 1; sex: juvenile; **Taxon:** scientificName: *Johngarthia planata* Stimpson, 1860; namePublishedIn: Stimpson W (1860) Notes on North American Crustacea, in the museum of the Smithsonian Institution, No II. Annals of the Lyceum of Natural History of New York 7: 176–246.; order: Decapoda; family: Gecarcinidae; genus: Johngarthia; specificEpithet: planata; scientificNameAuthorship: W. Stimpson 1860; **Location:** waterBody: East Pacific; country: Mexico; stateProvince: Oaxaca; verbatimLocality: Zapotengo Beach; **Identification:** identificationID: Robert Perger; **Event:** year: 2011; habitat: beach; **Record Level:** type: Photograph; rightsHolder: Claudia Glechner

## Analysis

**Figure 1.**
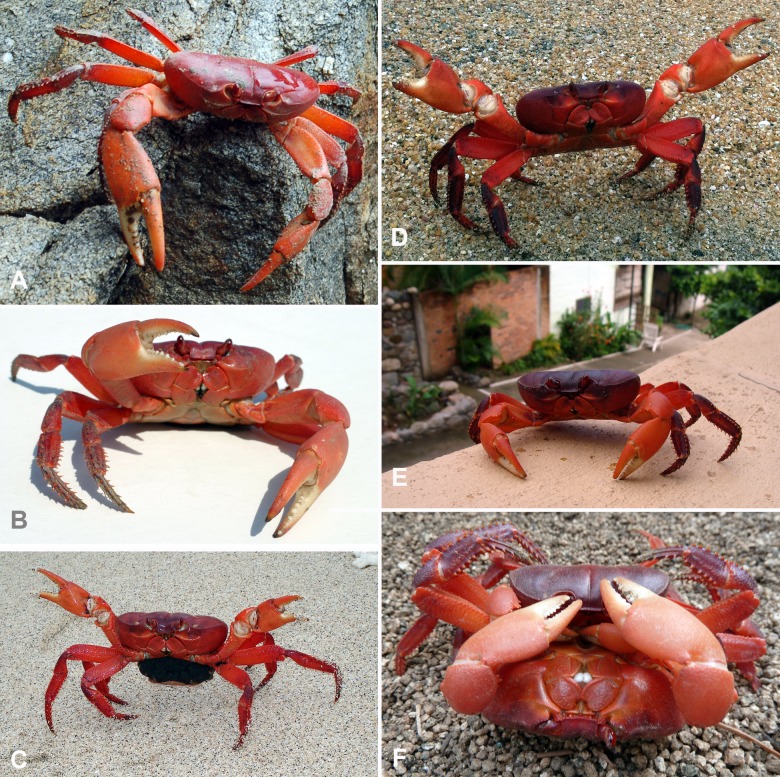
*Johngarthia planata* (Stimpson, 1860) from continental mainland of Mexico and Iguana Island, Panama. A, Mexico, Oaxaca, Escondido Beach (photo by Mike Gardiner, 2010); B, Mexico, Colima, Manzanillo (photo by Derek Zoebelein, 2004); C, Panama, Iguana Island (photo by María Elena Valencia, 2006); D–E, Mexico, Nayarit, Sayulita (photos by Madeline Milne, 2013; Tania Beagley-Brown, 2007; and Daniel Brewer, 2011, respectively).

**Figure 2.**
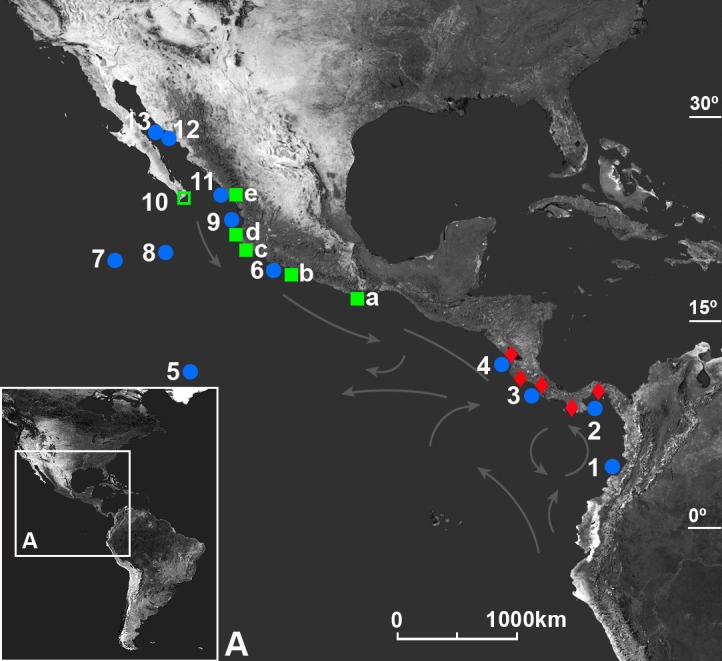
Distribution of *Johngarthia planata* (Stimpson, 1860): (●) island records; (□) peninsula records; (■) mainland records; (♦) locations with confirmed mainland absence of *Johngarthia planata* (Perger et al. 2013; Perger unpubl. data). Colombia: (1) Gorgona Island. Panama: (2) Iguana Island. Costa Rica: (3) Caño and Nairita Islands; (4) Colorada Island. Mexico: (5) Clipperton Island; (6) Michoacán, Pajaro Island; (7) Clarion Island; (8) Socorro and San Benedicto Islands; (9) Maria Cleofa and Isabela Islands; (10) Baja California Peninsula, Cabo San Lucas; (11) Sinaloa, Pajaro Island; (12) Sonora, Bacochibampo Bay; (13) Gulf of California, San Pedro Nolasco Island; (a) Escondido, Mazunte, and Zapotengo Beach, Oaxaca; (b) Ixtapa, Guerrero; (c) Manzanillo, Colima; (d) Sayulita, Nayarit; Mismaloya, Jalisco; (e) Mazatlán, Sinaloa. Major sea currents indicated by arrows (modified after Kessler 2006).

The Internet search revealed photographs of 12 individuals from Mexico (mainland) and three individuals from Panama (Iguana Island) (Table 1, Figs 1, 2). The diagnostic characters used (both apical lobes of the third maxilliped merus subequal, the leg spines prominently developed, the carapace depressed and the dark red color with orange chelipeds and cream cutting edges) (Fig. 1) were consistent with other examined specimens found on islands in Mexico and Costa Rica (Perger et al. 2013) as well as on Gorgona Island, Colombia (see Shih 2013). The specimens found on the Mexican mainland are the first continental mainland specimens ever recorded, and the specimens on Iguana Island are the first recorded in Panama. The species has been previously reported by Bright and Hogue (1972) and Jiménez et al. (1994) for “Panama” in error, likely following the reference “Malpelo Island, off Bay of Panama” by Rathbun (1918). Malpelo Island is inhabited by *Johngarthia malpilensis*, but the island is part of Colombia.

All mainland records of *Johngarthia planata* cited here refer to tourist beaches close to villages or small cities. Several photographs show individuals of *Johngarthia planata* foraging diurnally, running along house walls or even inside of houses and mating (Fig. 1).

The Internet search did not reveal any photographs of *Johngarthia planata* from Guatemala, El Salvador, Nicaragua and Costa Rica. For the latter country, the only known records to date are those cited by Perger et al. (2013).

## Discussion

Paulay and Starmer (2011) attribute the absence of land crab species on the continental mainland to the presence of predators. However, this pattern is less pronounced in the Neotropics where *Cardisoma crassum*, *Cardisoma guanhumi*, *Gecarcinus lateralis* and *Gecarcinus quadratus* share evolutionary traits which allow it to adapt to the conditions in a coastal band of the continental mainland (e.g. Bright 1966, Sherman 2002, Lindquist et al. 2009). On the other hand, the remaining four species of *Johngarthia* (see López-Victoria and Werding 2008, Hartnoll 2010, Perger et al. 2011) and *Gecarcinus ruricola* (Bright and Hogue 1972) have been reported from islands only, supporting the pattern suggested by Paulay and Starmer (2011). Several studies strongly indicate that *Johngarthia planata* has not established populations along the Central American mainland coast (see Perger et al. 2013 for a short review; Fig. 2), which agrees with the pattern of mainland absence observed in the four congeners. The ecological flexibility of *Johngarthia planata*, which occurs in habitats ranging from island rainforests to rocky terrain with sparse vegetation (Pérez-Chi 2005, Perger et al. 2013), suggest that the most significant factors which preclude such species from inhabiting the mainland are indeed competition or/and predation (Perger et al. 2013). The presence/absence pattern of *Johngarthia planata* in continental mainland habitats roughly coincides with the replacement of the moist forests of Panama and Costa Rica by dry forests that extend from Nicaragua to Mexico (see Ricketts et al. 1999). The diversity of possible crab predators such as birds (Orme et al. 2005) and mammals (Ceballos and Ehrlich 2006) is lower in the Southern Pacific Dry forest ecoregion than in tropical rainforests of Central America. Whether *Johngarthia planata* appears as well in non-urban areas along the Mexican Pacific coast remains unclear, however, the mainland establishment of such species might be facilitated close to or in urban areas where the lower diversity or/and abundance of possible predators/competitors is influenced by the presence of humans. Studies from undisturbed forest in the Pacific coast of Costa Rica (Sherman 2002, Griffiths et al. 2007, pers. observ.) relate cryptic behavior of gecarcinid land crabs to less suitable abiotic conditions and predator presence. On Caño and Nairita (Perger et al. 2013), Maria Cleofa (Rathbun 1899), Socorro (Pérez-Chi 2005) and Gorgona (López-Victoria and Werding 2008) islands, *Johngarthia planata* is active at night, which is most likely due to the presence of bird predators. In contrast, observations from Sayulita (Jolley and Brewer 2010), a newly reported mainland record for *Johngarthia planata* (Table 1; Fig. 1), suggest that the land crab activity there is less influenced by predator impact:

“*Visitors who arrive in Sayulita during the rainy season (June-October) and decide to take a leisurely walk through the jungle towards the beach are usually quite surprised, even shocked, to find the jungle floor literally teeming with thousands of purple-and-yellow and red land crabs. Crabs seem to be foraging everywhere in the leaf litter, wildly waving their claws and scurrying away to their burrows and caves at the first apprehension of danger. Most people find them interesting and amusing, even comical, with their tubular, hyper-vigilant eyes, their defensive postures and wild gestures*.”

Further photographs and accounts by Jolley and Brewer (2010) and Brewer (in litt.) indicate that *Johngarthia planata* is a common element of the urban wildlife and seeks food and shelter in closest proximity to human houses:

“*People who have built homes in the jungle near the ocean, however, consider the all-pervasive land crabs quite troublesome. If the crabs can get into the house (they can climb walls!), they can damage clothing, books, and food. Outside of the house, they are quite hard on garden plants, shredding foliage and damaging plant roots from inside their tunnels. By the time November comes, most folks are happy to see the crabs retreat into the ground!*” (Jolley and Brewer 2010)

These observations demonstrate that human presence does not necessarily have a negative impact on land crabs, as suggested by Paulay and Starmer (2011). The relationship between land crabs and humans might depend on the type of habitat, human settlement system and land use. Exploitation by hunter-gatherers on islands and the introduction of domesticated animals are assumed to have resulted in the extinction of local populations of *Gecarcinus ruricola* (Pregill et al. 1988) and a species of *Geograpsus* (Paulay and Starmer 2011). However, the consumption of land crabs has not been reported from tourist destinations along the Neotropical Pacific coast. As free-roaming domestic animals may be seen as a detriment to tourism (Webster 2013), populations of possible land crab predators/competitors such as pigs, cats, dogs and rats (see Paulay and Starmer 2011) tend to be more controlled than in areas with farmers or hunter-gatherers.

Because gecarcinid land crabs significantly influence tree recruitment by consuming seeds, propagules and seedlings, they are considered important ecosystem engineers (reviewed by Lindquist et al. 2009). While human presence might open a new evolutionary pathway for these otherwise island restricted land crabs, the colonization of continental mainland forests by a large species such as *Johngarthia planata* (see López-Victoria and Werding (2008) for a size comparison) would certainly have profound consequences for the structure and species composition of coastal ecosystems, particularly in view of the possibility that their foraging activity would be less restricted due to lower predation pressure. Further surveys for *Johngarthia planata* and possible crab predators/competitors are needed to determine if the known distributional pattern of such species is the result of human influence.

## Supplementary Material

XML Treatment for Johngarthia planata

## Figures and Tables

**Table 1.** Island and mainland records for *Johngarthia planata* obtained from the literature (L) and photographs (P); new records highlighted by (*).

Location	Source
**Colombia**	
Gorgona Island	Prahl 1983 (L)
**Costa Rica**	
Colorada Island	Perger et al. 2013 (L)
Caño Island	Perger et al. 2013 (L)
Nairita Island	Perger et al. 2013 (L)
**Panama**	
Iguana Island*	María Elena Valencia 2006; Julio Larish 2013 (P)
**Mexico, islands**	
Clipperton Island	Lenz 1901, Rathbun 1918, Garth 1965 (L)
Pájaros Island, Michoacán	García-Madrigal 2000 (L)
Isabela Island, Nayarit	Rathbun 1899 (L)
Maria Cleofa Island, Nayarit	Rathbun 1899 (L)
Pájaros Island, Sinaloa	Arzola-González et al. 2010 (L)
San Pedro Nolasco Island, Gulf of California	Felger et al. 2011 (L)
Socorro Island	Rodriguez et al. 1996, Ortega-Rubio et al. 1997 (L)
San Benedicto Island	Brattstrom and Howell 1956 (L)
Clarion Island	Hernández-Aguilera et al. 1986 (L)
Bacochibampo Bay, Sonora (small reef)	Manrique 1981 (L)
**Mexico, Baja California Peninsula**	
Cabo San Lucas	Stimpson 1860 (L)
**Mexico, mainland**	
Mazatlán, Sinaloa*	Lisa Brettschneider 2008 (P)
Sayulita, Nayarit*	Lisa Johnston 2005; Tania Beagley-Brown 2007; Daniel Brewer 2010; Madeline Milne 2013 (P)
Mismaloya, Jalisco*	Darin Williams 2007 (P)
Manzanillo, Colima*	Derek Zoebelein 2004 (P)
Ixtapa, Guerrero*	Miguel Angel Morales 2012 (P)
Mazunte, Oaxaca *	Gustavo A. Zambrano Cabrera 2008 (P)
Escondido Beach, Oaxaca*	Mike Gardiner 2010 (P)
Zapotengo Beach, Oaxaca*	Claudia Glechner 2011 (P)
